# Can thyromental distance be measured accurately?

**DOI:** 10.1007/s10877-017-0090-3

**Published:** 2017-12-12

**Authors:** Bin Wang, Hui Peng, Weidong Yao, Ling Guo, Xiaoju Jin

**Affiliations:** 1 Department of Anesthesiology, Wannan Medical College First Affiliated Hospital, Yijishan Hospital, Wuhu, China; 2 Department of Hospital Infection Management, Wannan Medical College First Affiliated Hospital, Yijishan Hospital, Wuhu, China

**Keywords:** Airway management, Thyroid cartilage, Anatomic landmarks, Accuracy, Ultrasonography, Volunteers

## Abstract

Using the thyromental distance (TMD) measured based on the ultrasonographic location of the thyroid cartilage prominence as the criterion, we investigated the accuracy of TMD measurement by surface landmark identification of the thyroid cartilage prominence. Twenty-nine anesthetist resident volunteers were recruited, including 10 first-year residents, 9 second-year residents and 10 third-year residents. Each volunteer measured the other 28 volunteers’ TMD. Then, the thyroid cartilage prominence of each volunteer was identified by ultrasonography of the junction of the vocal cord and thyroid cartilage, and the TMD was measured precisely. The error of the TMD measurement was determined by the minimal detectable difference (MDD) compared to the ultrasound measurement. A difference of greater than 5.4 mm between the TMD measured by volunteers and that based on ultrasound localization was defined as a measurement error. The measurement error rate of females’ TMD was significantly higher than that of males’ (50 vs 10%, *P* < 0.001). The error rates of anesthetist residents of first-year, second-year and third-year were 34, 27, and 31%, respectively, and were not significantly different. The error of TMD measurement by surface landmark identification is often, especially for women. More clinic experience don’t improve it.

## Introduction

Managing a difficult airway remains a significant problem in anesthesia and threatens patient safety during the preoperative period [1–4]. The accurate and rapid prediction of a difficult airway would greatly facilitate airway management. The thyromental distance (TMD), which is measured along a straight line from the thyroid cartilage prominence to the lower border of the mandibular mentum with full head extension, is a common method to predict difficult airways [5]. The smaller the TMD, the greater the probability of a difficult airway [6, 7]. However, the reported predictive values vary greatly. The sensitivity of the TMD varies from 15 to 95%, and the specificity of the TMD varies from 24 to 98% [8–11]. The cut-off points of TMD also differ greatly. Most scholars suggest that the cut-off point should be 6.5 cm in a normal adult [12–14], whereas others have considered cut-off points of 7.0 cm [7], 6.0 cm [15], 5.5 cm [16] and even 4 cm [10].

Studies have shown that localization of the front of the neck landmarks may be prone to error. The accuracy of the positioning of the cricothyroid membrane is very low [9, 17]. The cricothyroid membrane was accurately identified by digital palpation in 25–71% of normal subjects [17, 18, 24]. The cricothyroid membrane was accurately identified with digital palpation in only 0–39% in obese women due to the less prominent thyroid cartilage and excessive adipose tissue in the neck [17–19].

The accuracy of the positioning of the thyroid cartilage prominence and its potential influence on the predictive value of the TMD remain unclear. If measurement errors do occur in evaluating TMD, how high is the error rate? Consequently, the purposes of this study were as follows: (1) to investigate the accuracy of TMD measurements in the general population and the error rate; (2) to investigate whether there is a difference between men and women in TMD measurement errors; and (3) to determine whether the accuracy of anesthetist residents increases with learning stage.

## Methods

Approval was obtained from the ethics committee of Yijishan Hospital of Wangnan Medical College, and written informed consent was obtained from all volunteers.

Twenty-nine anesthetist resident volunteers were recruited, including 10 first-year resident volunteers with more than 6 months of clinical anesthesia practice, 9 second-year resident volunteers with more than 1 year of clinical anesthesia practice, and 10 third-year resident volunteers with more than 2 years of clinical anesthesia practice. These volunteers included 14 male volunteers and 15 female volunteers. The major of all volunteers is anesthesia. All of them were familiar with evaluations of TMD. The training of thyromental distance measurement was contained at the training of difficult airway evaluation before they went into clinic work.

To avoid disagreement on the upper bound of the TMD, the lower border of the mandibular mentum of all volunteers was marked with a marker pen. Each volunteer was assigned a straight millimeter ruler and then asked to measure the TMD [11] of the other 28 volunteers and record the results privately. All volunteers sat with full head extension as his/her TMD was measured. To avoid affecting the measurements of others, visual marks were not made on the thyroid cartilage prominence. All measurements were performed separately, with no communication between the volunteers. No one of TMD measurement was more than 1 min once time. Finally, the TMD of each volunteer was identified with ultrasound by one experienced anesthesiologist who was experienced in ultrasonography.

**Fig. 1:**
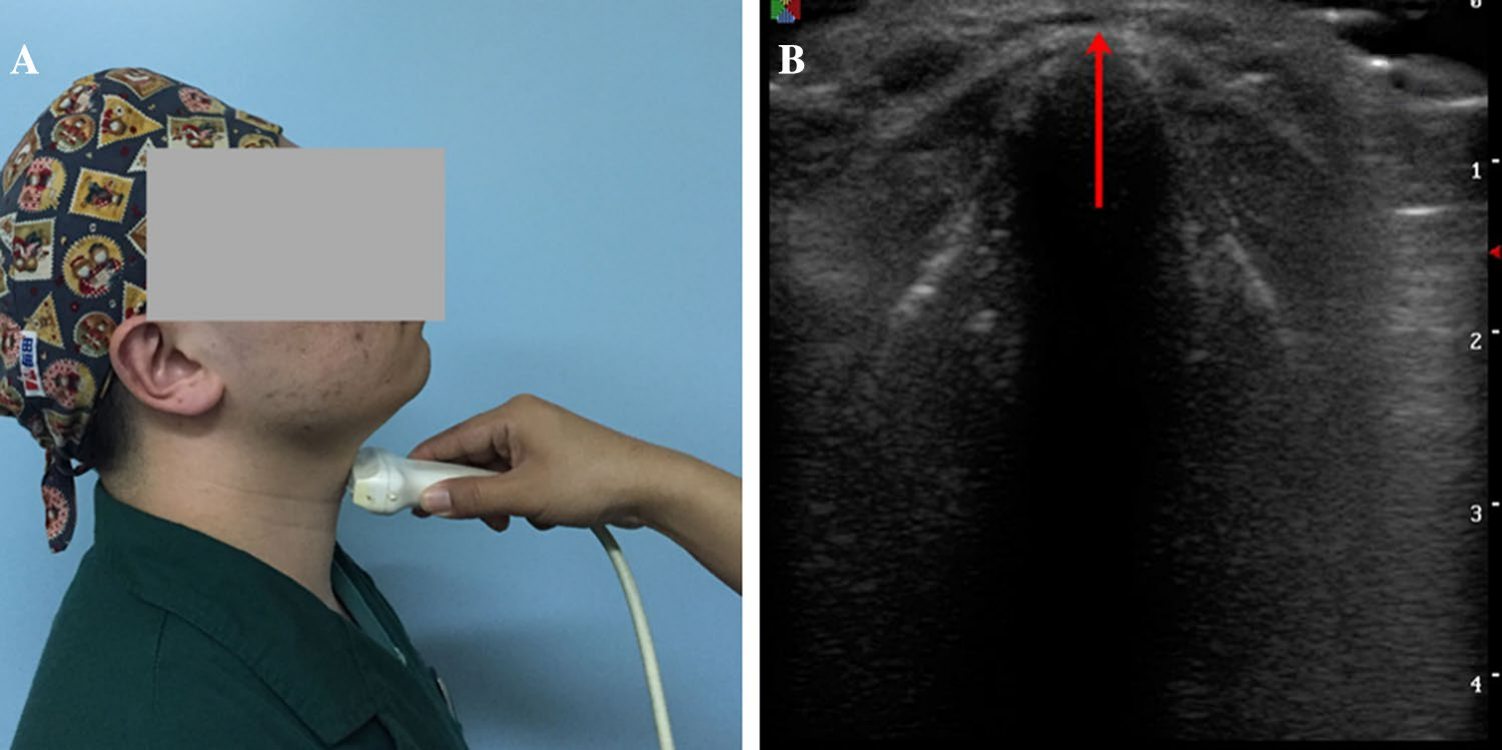
The methods used to locate the thyroid cartilage prominence. The ultrasound transducer probe was placed horizontally and moved upward to reveal the junction of the vocal cord and thyroid cartilage (indicated by the arrow)

The thyroid cartilage prominence of each volunteer was identified by ultrasound. The ultrasonography of cases included in the study was performed by one of the examiners, who was an experienced sonographer, using a portable ultrasound machine (S8, SonoScape Corp LP, Shenzhen, China) with a high-frequency linear probe. The thyroid cartilage prominence was located as follows. The volunteer sat with full head extension and was asked to relax and not swallow. A high-frequency linear transducer probe was horizontally placed on the annulus tracheae above the suprasternal fossa in the midline horizontal plane. The probe was moved up to show the vocal cord clearly on the screen, followed by the junction of the vocal cord and thyroid cartilage (anterior commissure), as shown in Fig. 1. Then, the junction position was marked on the skin surface with a marker pen. The upper end of the anterior commissure is very close to thyroid prominence. Whether in men or women, the position between them is relatively fixed [20]. So, TMDs were measured precisely along a straight line from the thyroid cartilage prominence located by ultrasound to the lower border of the mandibular mentum marked previously with full head extension. The person who performed the sonographic measurements was blinded to the results of the TMD measurements of volunteers.

The random variation of the TMD measurements was determined by calculating the minimal detectable difference (MDD) [21, 22]. Two examiners with 3 years of experience in sonographic assessment measured 40 volunteers’ TMDs by ultrasound with the methods described above. Descriptive data are reported as the mean ± standard deviation. The MDD was calculated using the following formula:

$${\text{MDD}_{\%}}={\text{z}} \times {\text{SEM}} \times \sqrt {\text{2}} .$$

The standard deviation (SEM) was 1.94 mm. The MDD_95%_ in the current study was 5.4 mm. Thus, 95% of stable volunteers in this population would be expected to show a random variation of < 5.4 mm in repeated trials. In this experiment, we selected 5.4 mm as the threshold of errors in TMD measurements. When the difference between the measurement using surface landmark identification and the measurement based on ultrasound location of the thyroid cartilage was ≥ 5.4 mm, it was defined as a measurement error.

According to the value of the TMD based on ultrasound, the thyroid cartilage prominence was located to determine whether the volunteers’ TMD measurements were correct.

Primary outcomes: TMD measurements’ error rates in males and females who were measured, and in different years resident volunteers when they performing the measurements were calculated.

Secondary outcomes: the differences of TMDs between the measurement using surface landmark identification and the measurement based on ultrasound location of the thyroid cartilage.

### Statistics

Continuous variables, such as the height, weight, Body Mass Index (BMI) of the volunteers and TMD are presented as the mean ± standard deviation (SD). The results for male and female volunteers were compared with Student’s *t* test. One-way analysis of variance was used to analyze the differences among different anesthetist residents at different learning stages. If the data were not normally distributed, non-parametric tests were applied for continuous variables. Categorical variables, such as the rate of errors in TMD measurements, were compared using the *x* ^2^ test or Fisher’s exact test. All statistical tests were two-sided tests (test level α = 0.05), and *P* values < 0.05 were considered statistically significant.

## Results

A total of 29 volunteers participated in the TMD measurement study. The characteristics of the volunteers are shown in Table 1. The TMD of females was smaller than that of males.

**Table 1:** The characteristics and the measurement error rate of the thyromental distance of the volunteers who participated in the study

Variables	Male volunteers (*n* = 14)	Female volunteers (*n* = 15)
Age (year)	25.9 ± 2.2	26.0 ± 2.9
Height (cm)	174.3 ± 5.7	161.0 ± 4.0
Weight (kg)	65.2 ± 8.7	53.3 ± 8.1**
BMI (kg/m^2^)	21.4 ± 2.0	20.5 ± 2.3
BMI < 20/20–25/> 25 (kg/m^2^)	3/10/1	9/5/1
TMD by ultrasound	85.4 ± 10.7	71.6 ± 7.6**
Overall measurement times	392	420
Measurement error times	39	212
Error rate (95% CI)	10% (7–13%)	50% (46–55%)**

TMD measurements’ error rates in males and females who were measured were calculated. Continuous variables, such as the age, height, weight, Body Mass Index (BMI) of the volunteers and TMD are presented as the mean ± standard deviation (SD). Measurement times and BMI < 20/20–25/> 25 (kg/m^2^) were represented by instance numbers. The error rate was expressed as a percentage

*CI* confidence interval

**Compared to male volunteers, *P* value < 0.01

Primary outcomes: of a total of 812 measurements, 251 measurements exhibited measurement error. The error rate was 10% for males and 50% for females. There was a significant difference in the accuracy of the TMD between male volunteers and female volunteers. The measured error rate of the females was significantly higher than that of the males, as shown in Table 1.

The measurement accuracy of the TMD among anesthetist residents at different learning stages by surface landmark identification with a millimeter ruler was 34, 27, and 31%, respectively. These values were not significantly different. The results are shown in Table 2.

**Table 2:** The measurement accuracy of the thyromental distance by anesthetist residents at different learning stages by surface landmark identification

	*n*	Measurements of each	Measurements overall	Measurement errors overall	Error rate (95% CI)
First-year residents	10	28	280	94	34% (28–39%)
Second-year residents	9	28	252	69	27% (22–33%)
Third-year residents	10	28	280	88	31% (26–37%)

*CI* confidence interval

**Fig. 2:**
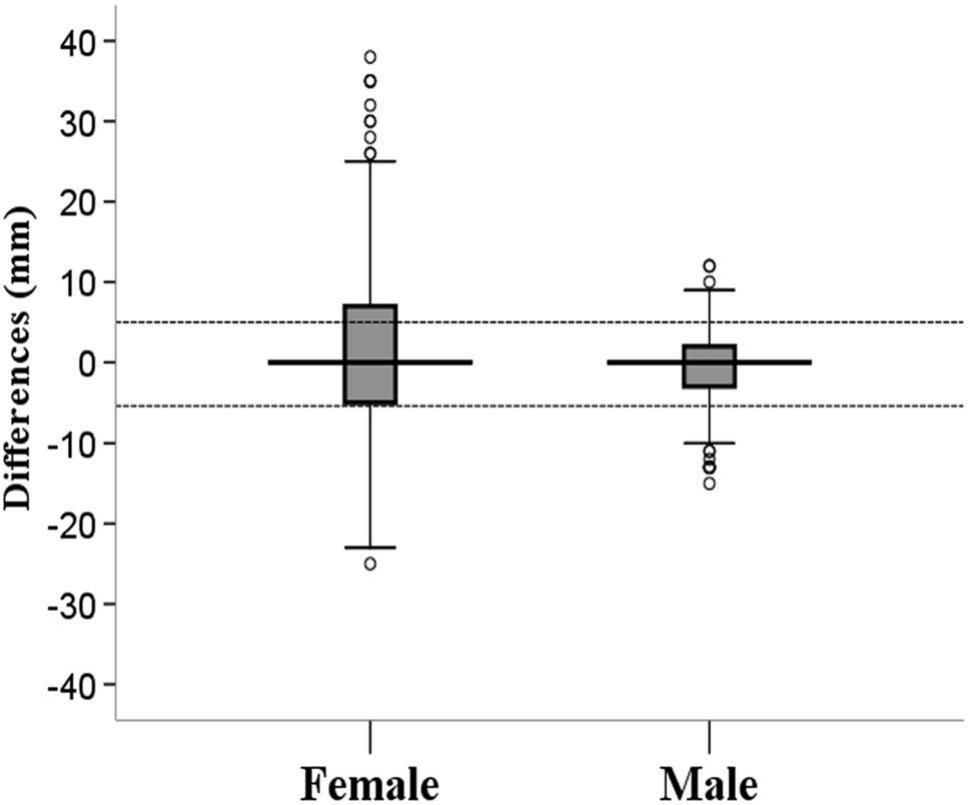
The differences of TMDs between the measurement using surface landmark identification and the measurement based on ultrasound location of the thyroid cartilage. The dotted line indicates the difference is 5.4 mm. Among values of the differences, the upper and lower quartiles constituted the two horizontal sides of the box, two short dashes at the ends of the box were minimum and maximum value excluding abnormal value. The long line in the middle of box was median value. Small hollow circles mean the abnormal values > 1.5 times the four percentile interval

Secondary outcomes: the differences of TMDs between the measurement using surface landmark identification and the measurement based on ultrasound location of the thyroid cartilage are shown in Fig. 2. The average absolute deviation of women’s rank is higher than that of men’s, mean rank is 512 of the female and 323 of the male.

## Discussion

The error rate of TMD measurements was high, especially in females. The error rate for males was 10%. For females, the error rate was higher, up to 50%, and the differences of TMDs between the measurement using surface landmark identification and the measurement based on ultrasound location of the thyroid cartilage of the females were significantly greater than that of males. This discrepancy may be due to the relative difficulty of locating the thyroid cartilage prominence in females. In addition to the thyroid cartilage, front neck landmarks include the hyoid bone, cricoid cartilage, and tracheal cartilages. Because the female TMD measurement error rate was obviously higher than the male error rate, and the average absolute deviation of women’s rank is higher than that of men, we deduced that the cricoid cartilage, tracheal cartilages and even the hyoid might be mistaken for the thyroid cartilage prominence in females. If the cricoid cartilage or tracheal cartilages are mistaken for the thyroid cartilage prominence, the numerical value of the TMD will increase significantly. If the hyoid bone is mistaken for the thyroid cartilage prominence, its value will decrease significantly. We observed no significant differences between the measurements made via surface landmark identification and the measurements made via ultrasound localization of the TMD. We deduced that two types of errors occur in the measurement of TMD in clinical practice.

Other factors that might cause measurement error include differences in head extension, pouting or pursing of the lips, and the positioning of the thyroid cartilage prominence. Although TMD measurement requires extension of the head as far as possible, the degree of head extension may differ. To avoid localization errors in the lower border of the mandibular mentum, we unified the border. However, the skin clearly lifts when pouting or pursing one’s lips, leading to a measurement difference. The marker line used in the localization of the mandibular mentum border also has a certain width itself, which may also lead to measurement differences. Some people may use the upper bound of the marker line, while others may use the lower bound. Although the thyroid prominence is obvious and easy to identify in males, the thyroid prominence is not a point but a relatively small area. Thus, its measured location may vary.

Many studies have used ultrasound to locate neck structures [17–21, 23, 24]. Therefore, in this experiment, we located the thyroid cartilage prominence by ultrasonography. By ultrasound localization, TMD can be measured accurately and avoid positioning error. We hope that the prediction value of difficult airway would increase when TMD measured by ultrasound. However it needs further investigation.

Interestingly, the accuracy of TMD measurements did not improve with prolonged learning time, which was similar to some airway management research [25], simulation-based training may be not superior to non-simulation based training on airway management training. In this experiment, there were no significant differences in accuracy among first-year residents, second-year residents and third-year residents. This lack of difference may be because the measurement of surface landmarks with a ruler is simple and easy to master. However, the surface landmark of the thyroid cartilage prominence is not obvious and can be difficult to locate, especially in women. Therefore, it is very important to develop effective methods to position the thyroid cartilage. The ultrasound method used to locate the thyroid cartilage prominence in this experiment is accurate and easy to learn. It may thus be an effective tool to measure the TMD.

The prediction value of the TMD depends on accurate measurement. There are two common methods for clinically measuring the TMD: measurement by finger width and measurement by ruler. A TMD less than three finger widths is considered a difficult airway, but the actual width of three fingers can fluctuate from 4.6 to 7.0 cm (mean 5.9 cm) and varies between the proximal and distal interphalangeal joints and between genders. Baker et al. [11] found that the finger measurement of thyromental distance has little clinical significance. Measurement by surface landmark identification is significantly more accurate than finger measurement. The clinical value is limited to improving the prediction accuracy of the TMD of difficult airways by adjusting the cut-off point. Some scholars believe that the TMD is useless for predicting a difficult airway [9]. Our study further confirmed that the error of TMD measurement by surface landmark identification is also often. We doubt that the prediction value of the TMD may be limited by the inability to measure the TMD accurately. Therefore, is it necessary to measure the TMD more accurately?

There are some limitations in present study. The sample size of this study is small. Volunteers in this study did not cover the age groups of the entire anesthesiologist, such as the middle-aged and the elderly. We only studied residents of 3 years, this will limit the conclusions of this study generalize to other groups of anesthesiologists. This study also lacks data on the accurate measurement of thyromental distance in the prediction of difficult airways. What’s more, in some subjects, neck movements is limited in clinic practice, and is critical in the prediction of difficult airways. If the mentum anatomy had not been pre-defined in our research, the error would like to be greater. It seems likely that more obese subjects would have lead to greater error. However, all volunteers take part in our research were young people, few with obese, and only two BMI > 25 kg/m^2^. The data in our research would be surrogates for more important outcomes.

## Conclusions

TMD measurement errors generally exist. TMD measurement error rate for women is higher than that for men. The accuracy of anesthetist residents dose not increase with learning stage. Whether accurate measurement of TMD can improve its prediction value needs to be further clarified.
